# Thrombus formation in the suprahepatic inferior vena cava after microwave ablation in patients with hepatic metastasis: a case report

**DOI:** 10.1186/s12959-023-00481-8

**Published:** 2023-04-04

**Authors:** Jun Ma, Juan Zhu, Tengyun Ding, Libin Cai, Chaoping Zhou, Yaming Zhang

**Affiliations:** 1 Department of General Surgery, Anqing Municipal Hospital, No. 352, Ren-Ming Road, Anqing, Anhui Province 24600 P.R. China; 2 Department of Imaging, Anqing Municipal Hospital, Anqing, 246000 P.R. China; 3 Department of Ultrasound, Anqing Municipal Hospital, Anqing, 246000 P.R. China

**Keywords:** Rectal cancer, Hepatic metastasis, Thrombus, Microwave ablation

## Abstract

**Background:**

Microwave ablation (MWA) via ultrasound guidance is an important tool in the treatment of liver metastases. The most common postoperative complications are abdominal hemorrhage and bile leakage, whereas thrombosis in the suprahepatic inferior vena cava (IVC) is very rare, and clinical management is very difficult when the head end of the thrombus reaches the right atrium.

**Case presentation:**

This is a case report of a 52-year-old man with hepatic metastasis 21 months after radical resection of rectal cancer. After chemotherapy combined with targeted therapy, metastasis in segment IV (S4) of the liver was treated with microwave ablation. Two months after treatment, the hepatic metastasis in S4 showed a microwave ablation zone on MRI.Enhanced MRI showed venous thrombosis located in the left hepatic vein and IVC, and the head of the thrombus reached the right atrium. After two weeks of anticoagulation and thrombolytic treatment, the follow-up MRI showed that the venous thrombus had nearly disappeared.

**Conclusion:**

When liver metastases are close to the hepatic vein, clinicians should pay attention to the occurrence of hepatic vein and IVC thrombosis following MWA; through early diagnosis and anticoagulation, pulmonary thromboembolism (PTE) can be minimized.

## Background

Approximately 50% of patients with colorectal cancer will suffer from simultaneous or metachronous liver metastases [1, 2]. Microwave ablation (MWA) is an important treatment for liver metastases, with the advantages of high safety, minimal trauma, and significant treatment effects [3]. The most common complications following MWA are abdominal hemorrhage, bile leakage, abnormal liver function and pleural effusion, whereas thrombosis in the suprahepatic inferior vena cava (IVC) is extremely rare [4, 5].

When thrombosis develops, the head of the embolus will be close to the right atrium and very difficult to handle. If the thrombus is dislodged, the patient may develop symptoms of pulmonary thromboembolism (PTE), which may be fatal. This case report presents a patient who underwent MWA for postoperative liver metastasis of rectal cancer. After treatment, thrombosis occurred in the left hepatic vein and suprahepatic IVC, but the patient’s thrombosis nearly disappeared through accurate diagnosis and rational treatment.

## Case presentation

A 52-year-old Chinese man presented with thrombus formation in the left hepatic vein and suprahepatic IVC 2 months after treatment of hepatic metastases by MWA.

**Fig. 1:**
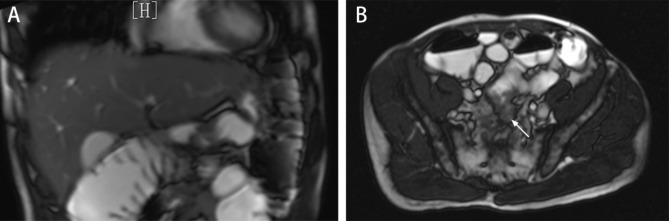
Preoperative imaging data A: MRI showed no metastatic lesions in the liver. B: MRI showed that the lesion was located in the lower part of the rectum(white arrow), and there was no lateral lymph node metastasis in the pelvis

In April 2020, the patient received a diagnosis of rectal adenocarcinoma. Magnetic resonance imaging (MRI) showed that the lesion was located in the lower part of the rectum, and there was no lateral lymph node metastasis in the pelvis **(**Fig. 1A**)**. CT showed no metastatic lesions in the liver **(**Fig. 1B**)**. The preoperative clinical stage was cT3N + M0. Preoperative neoadjuvant therapy was recommended, but the patient refused for economic reasons.

Laparoscope-assisted abdominal resection of rectal cancer (the Dixon procedure) was performed on April 14, 2020. Postoperative pathologic examination showed stage IIIA (pT4aN1cM0), moderately differentiated, KRAS-mutated (exon 4) adenocarcinoma with nodal involvement (0/13) and cancerous node formation in the rectal mesentery. The circumferential resection margin (CRM) was negative. Immunohistochemical markers were as follows: HER-2 (-), MSH2 (+), MSH6 (+), MLH1 (+), and PMS2 (+).

Between May and October 2020, the patient began adjuvant chemotherapy after surgery, receiving oxaliplatin and capecitabine (the CAPEOX regimen) for eight cycles. The patient refused postoperative radiotherapy due to economic reasons.

**Fig. 2:**
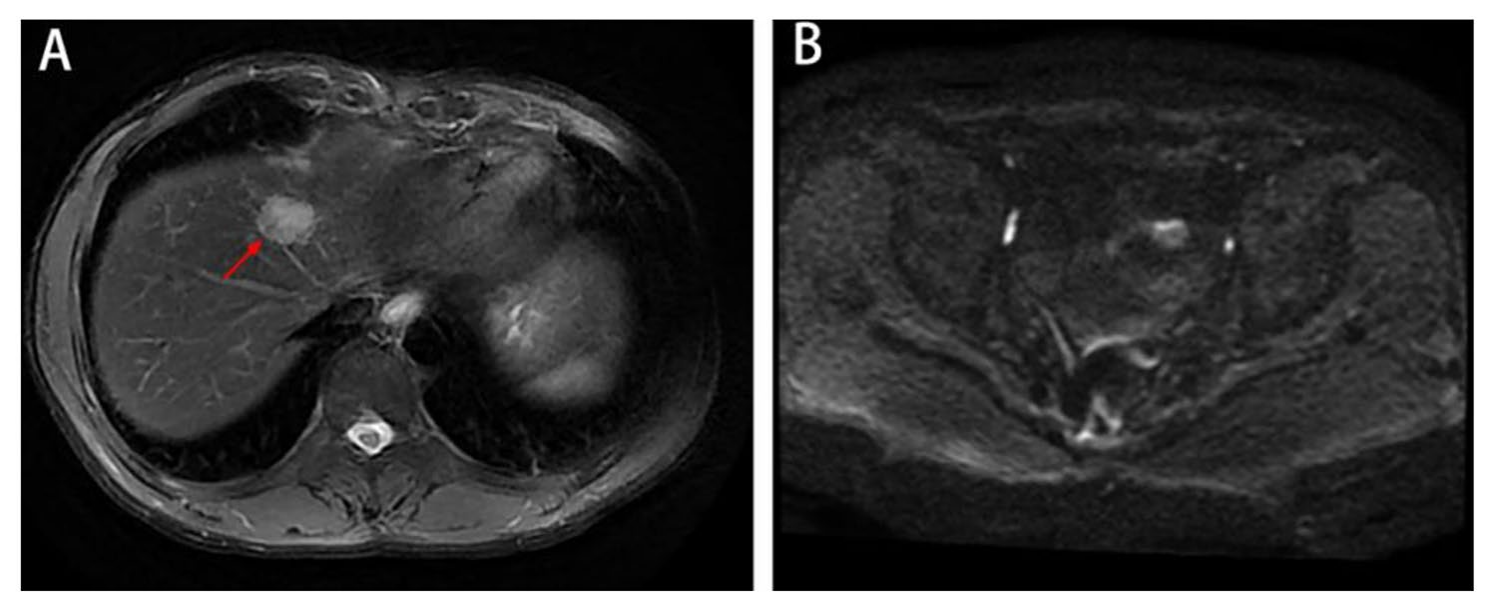
Imaging data from January 2022 A-B: MRI revealed a single metastatic lesion (2.5 cm) in the fourth segment of the liver, with no lesions in the pelvis

**Fig. 3:**
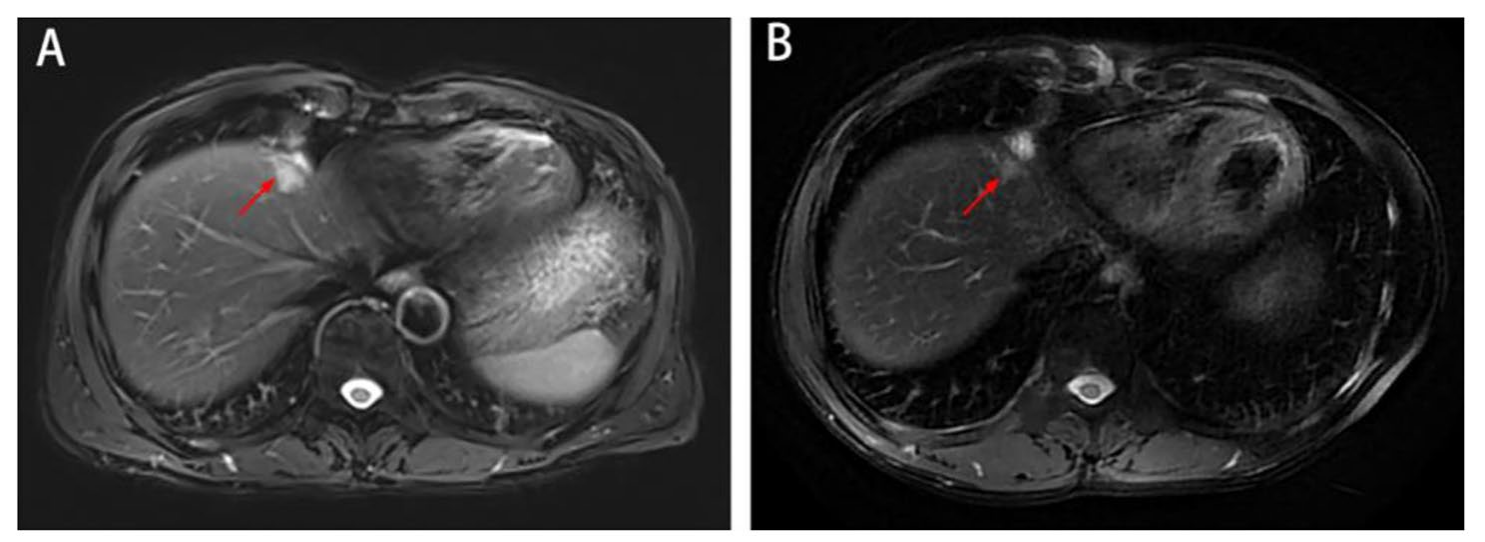
Liver metastatic lesions were significantly reduced after chemotherapy plus targeted therapy. A: In April 2022, MRI showed that the metastatic lesion measured 1.9 cm(red arrow). B: In June 2022, MRI showed a lesion (1.3 cm)in S4 of the liver (red arrow), and no new metastatic lesions were detected

In January 2022, MRI revealed a single metastatic lesion (2.5 cm) in segment IV(S4) of the liver, with no lesions in the lung or pelvis **(**Fig. 2A-B**)**. Between February 2022 and May 2022, a regimen of FOLFIRI plus bevacizumab was applied for 6 cycles. In April 2022, MRI showed that the metastatic lesion measured 1.9 cm **(**Fig. 3A**)**. In June 2022, MRI showed that the lesion (1.3 cm) in S4 of the liver was significantly reduced, and no new metastatic lesions were detected **(**Fig. 3B**)**.

**Fig. 4:**
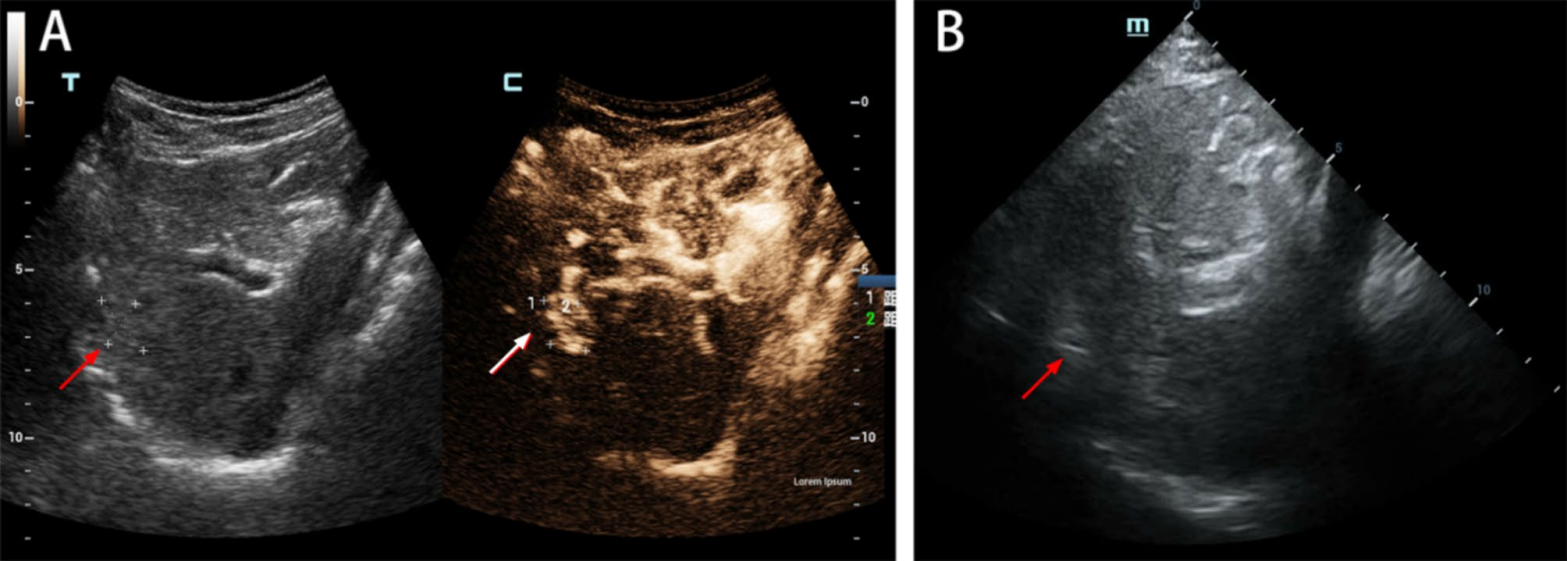
Intraoperative observations and the procedure for microwave ablation A: A medium-sized nodule of approximately 18 × 9 mm with poorly defined borders and uneven internal echogenicity (red arrow ) was visible in S4 on ultrasound. Ultrasonography showed enhancement of the lesion(white arrow). B: The hepatic mass in S4 was selected as the target for ablation(red arrow)

On June 27, 2022, after providing informed consent, the patient underwent ultrasound-guided MWA of the lesion. Intraoperatively, a medium-sized nodule of approximately 18 × 9 mm with poorly defined borders and uneven internal echogenicity was observed in S4 on ultrasound. A total of 2.0 mL of microbubble contrast agent was injected via the antecubital vein, and the lesion began to show enhancement at 24 s, began to fade at 45 s, and was completely cleared of the contrast agent after a delay period **(**Fig. 4A**)**.

The hepatic mass in S4 was selected as the target for ablation under general anesthesia **(**Fig. 4B**)**. Ultrasound-guided subdiaphragmatic artificial ascites placement was performed with a continuous saline drip to separate the diaphragm from the intestinal canal. The needle was then guided by ultrasound to the upper left posterior of the tumor in the lower right anterior abdomen. Subsequently, the ablation button was pressed. The ablation zone covering the tumor was not enhanced and was approximately 50 mm×25 mm in size.

After the treatment, the patient was in good general condition, with no nausea or vomiting, no dyspnea, no lower limb edema, normal eating and sleeping habits, and normal stool and urine.He had no history of hypertension, diabetes or heart disease and had no family history of cancer.His supraclavicular lymph nodes were not palpable, the abdominal incision was well healed, no abnormal masses were palpated in the abdomen, the rectal anastomosis was well healed, and the mucosa of the rectal wall was smooth.

Laboratory studies (reference ranges in parentheses) revealed the following. White blood cell count: 6000k/µL (4800–10,800k/µL), neutrophil count: 5400k/µL (2400–7200k/µL), platelet count: 196,000/µL (164,000-446,000/µL), total protein: 61 g/L (60–80 g/L), albumin: 42 g/L(40–55 g/L), hemoglobin: 13 g/dL (12–16 g/dL), C-reactive protein: 4.23 mg/L (< 6 mg/L), erythrocyte sedimentation rate: 5 mm/hr (< 20 mm/hr), D-dimer: 0.24 µg/mL (< 0.5 µg/mL), protein C: 106% (64–147%), protein S: 86% (78–124%), antithrombin III:90%(80-120%), anti-phospholipid antibodies: negative (negative), homocysteine: 11.5 µmol/L (< 15 µmol/L), and normal liver and kidney function tests.

The patient had no history of PTE or venous thromboembolism (VTE) and had no family history of thrombosis. According to the risk stratification method based on the Caprini score, the patient’s score of 3 corresponded to an intermediate risk of thrombosis. Considering the patient’s previous experience with bevacizumab and MWA therapy, early exercise and icosapent ethyl (IPE) were commended as antithrombotic prophylaxis.

**Fig. 5:**
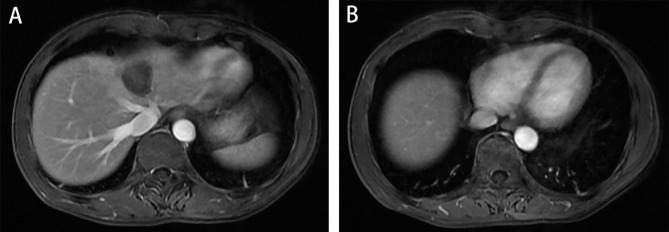
Imaging data from July 29th, 2022 A: MRI showed the S4 microwave ablation zone and left hepatic vein. B: There was no obvious thrombus in the suprahepatic IVC.

**Fig. 6:**
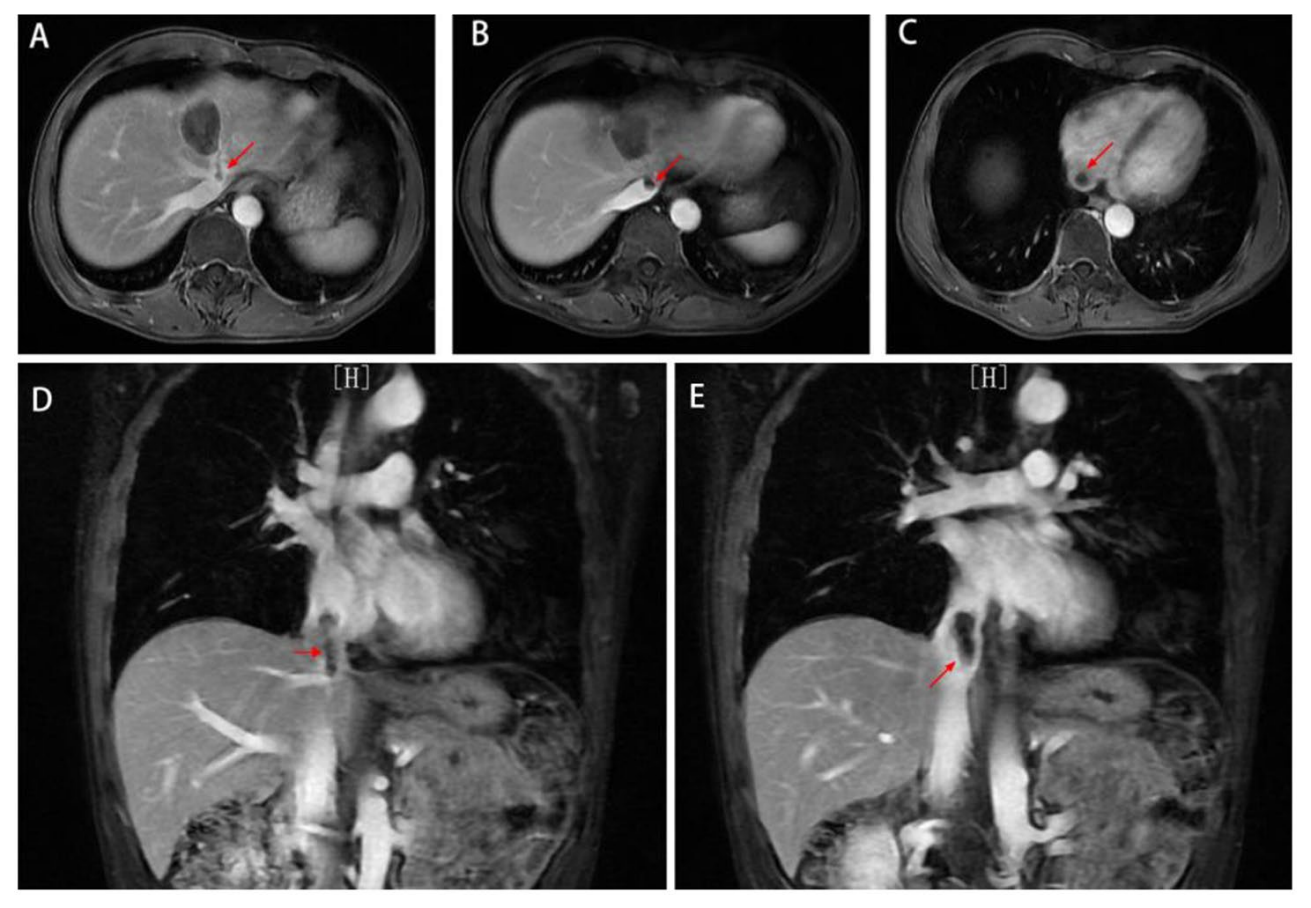
Imaging data from August 26th, 2022 A-E: MRI revealed the microwave ablation zone in S4; there was a thrombus(red arrow) in the left hepatic vein and IVC, and the head of the thrombus had entered the right atrium

On July 29, 2022, MRI showed an S4 microwave ablation zone, left hepatic vein visualization, and no obvious thrombus in the suprahepatic IVC **(**Fig. 5A-B**)**. On August 26, 2022, MRI revealed an S4 microwave ablation zone, thrombosis of the left hepatic vein and IVC, and extension of the head of the thrombus into the right atrium **(**Fig. 6A-E**)**.Factor V Leiden and PT20210 genetic mutation tests were recommended, but the patient refused for economic reasons.

**Fig. 7:**
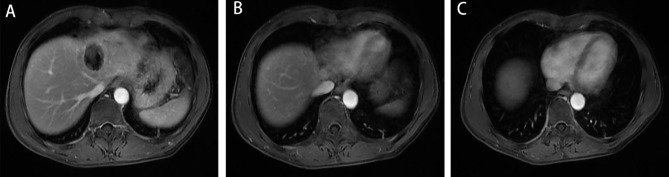
Imaging data from September 12th 2022 A-C: MRI showed that the thrombus in the left hepatic vein and IVC had essentially disappeared

**Fig. 8:**
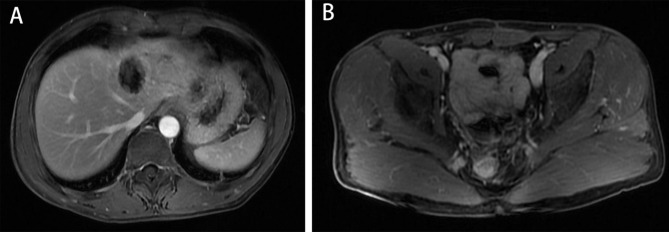
The latest imaging data A-B: MRI showed no metastatic disease or local progression

The patient was placed on bed rest and given anticoagulation therapy for two weeks (enoxaparin, 40 mg every 12 h). His blood count and coagulation function were closely followed. On September 12, 2022, MRI showed that the left hepatic vein and IVC venous thrombi had essentially disappeared **(**Fig. 7**)**. The patient continued to be treated with rivaroxaban (20 mg once a day) for three months and was in good general condition with no particular discomfort.No widespread metastasis or local progression was found in the latest follow-up **(**Fig. 8**)**.

## Discussion

Colorectal cancer (CRC) is the third most common cancer worldwide, and 25–35% of patients with CRC present with or developing colorectal liver metastases (CRLMs). Almost 20% of patients present synchronous CRLM, and 10–15% of patients present metachronous CRLM [6]. MWA is an important tool in the treatment of liver metastases and can achieve the same prognosis as surgery for a single lesion smaller than 3 cm [7].

More than 1 year after surgery for CRC, this patient presented with metachronous liver metastases. His lesion measured approximately 2.5 cm, and no local recurrence was observed. The patient was treated with chemotherapy plus targeted therapy, and the lesion shrank significantly (approximately 1 cm) but did not disappear completely, while no new lesions were found, which indicated that the time was optimal for local treatment. Surgical resection was recommended, but the patient refused surgery and was finally treated with percutaneous ultrasound-guided MWA.

In clinical practice, PTE is prevented by the placement of an IVC filter. However, a filter cannot be placed in the suprahepatic IVC because of its location near the right atrium. This complication is rare and has not been reported before. Despite the lack of relevant experience, we were able to successfully cure the patient through multidisciplinary discussions and a treatment plan of anticoagulation and breaking.

Vascular injury involves the intimal layer of the hepatic vein, leading to platelet aggregation and the subsequent formation of thrombosis [8]. The cause of thrombosis in this patient could be one of the following: (1) Chemotherapy can cause tumor tissue degeneration and necrosis, which affected the ultrasound imaging. During the operation, the needle was biased to the left side of the lesion, microwave heat caused a thrombus in the left hepatic vein, and the thrombus entered the IVC. (2) The tumor was high at the top of the diaphragm, where there was interference from pulmonary gas, which led to injury by the side of the tumor [9]. (3) Enhanced ultrasonography misdiagnosed the abnormal enhancement area as a metastatic lesion, which resulted in an extended ablation zone and caused hepatic vein injury.

This case provided insight to the authors due to the following observations. First, when the lesions are close to the hepatic vein, the formation of thrombi is a risk that requires close attention after MVA. One month after the procedure, no significant thrombus was detected in this patient, and a thrombus was observed only on re-examination 2 months after the procedure. If this complication had been missed, the dislodged clot might have lead to PTE. Second, for lesions at the top of the diaphragm, as well as lesions of small size, accurate localization is necessary, and intraoperative thoracic filling methods should be performed to reveal the lesions completely [10].

After MWA, clinicians are mainly concerned about common complications, such as bleeding, bile leakage, pleural effusion and lower extremity deep vein thrombosis (DVT), and are likely to ignore thrombosis located in the IVC. In our patient, the thrombus was identified mainly by MRI, and it had low intraluminal signal in the enhancement phase and a high signal in the T2-weighted phase.

We suspect that suprahepatic IVC thrombosis may originate from hepatic vascular thrombosis; therefore, it is important to focus on the formation of hepatic vein or portal vein thrombosis after radiofrequency ablation (RFA) or MWA. To our knowledge, the incidence of hepatic vascular thrombosis after RFA or MWA of liver tumors is low. We tried to identify the risk factors for liver thrombosis through a literature review. A summary of the literature on venous thrombosis following ablation of hepatic tumors is shown in Table 1 [11–30]. According to the literature, the incidence of thrombosis after radiofrequency ablation was 0.2% (6.2%), of which portal vein thrombosis was the most common, hepatic vein thrombosis was rare, and no cases of IVC thrombosis were found. The main risk factors for thrombosis included the following: (1) Tumors close to the vein. Vein thrombosis following RFA might be caused mainly by radiofrequency heat damage to the endothelial cells of the portal vein located near the tumor, which would lead to platelet aggregation and the subsequent formation of portal vein thrombosis [13, 15, 16, 18, 27]; (2) Liver cirrhosis. A cirrhotic liver might be more likely to have venous thrombosis than a noncirrhotic liver after RFA because of the relatively slow portal flow in cirrhotic livers [14, 15, 21, 22, 28]; (3) Hypercoagulative states. Preexisting thrombus, previous splenectomy, infection and malignancy cause hypercoagulative states and increase the probability of thrombosis in patients. [12, 16, 20, 28]; (4) The Pringle maneuver. It is routine practice to perform RFA without the application of Pringle maneuvers, and the resulting hepatic blood flow might help to protect the blood vessel against thermal injury from RFA by providing a ‘heat-sink’ effect [14, 17, 21]; (5) Vessels smaller than 3 mm in diameter. Thrombosis of vessels larger than 4 mm after RFA was infrequent, provided that normal flow was maintained through these vessels [14, 21]; (6) Percutaneous balloon occlusion (PBO). PBO can decrease hepatic inflow or outflow through a mechanical maneuver and lead to slow blood flow [19].

**Table 1:** Published cases of hepatic vascular thrombosis after RF or MWA of primary or secondary hepatic tumors

Author	Publication year	Country	Age (y) /sex	Primary or secondary hepatic tumors	Number(incidence of thrombosis)	Thrombosed vein	Time interval (d)	Associated thrombosis risk factors
Catalano et al. [11]	2000	Italy	NA	Primary	2(6.2%)	Portal vein	NA	NA
Francica et al. [12]	2000	Italy	53/M	Secondary	1(NA)	Portal vein	7	Hypercoagulative states
Ng et al. [13]	2002	China	43/M	Primary	1(NA)	Portal vein	14	Tumors close to the vein
Baère et al. [14]	2003	France	NA	Primary or secondary	11(3.5%)	Portal vein or hepatic vein	NA	1. Pringle maneuvers 2. Cirrhotic livers 3. Vessels smaller than 3 mm in diameter
Zheng et al. [15]	2003	Japan	78/M	Primary	1(NA)	Portal vein	6	1. Tumors close to the vein 2. Cirrhotic livers
Akahane et al. [16]	2005	Japan	NA	Secondary	4(0.6%)	Portal vein	NA	1. Hypercoagulative states 2. Tumors close to the vein
Jansen et al. [17]	2005	Netherlands	NA	Primary or secondary	2(1.6)	Portal vein	4/7	Pringle maneuvers
Ng et al. [18]	2005	China	NA	Primary	3(1.9)	Portal vein or hepatic vein	NA	Tumors close to the vein
Baère et al. [19]	2008	France	NA	Primary or secondary	9(4.5)	Portal vein or hepatic vein	NA	PBO
Orlacchio et al. [20]	2010	Italy	84/M	Primary	1(NA)	Portal vein	7	Hypercoagulative states
Kim et al. [21]	2011	Korea	NA	Primary	15(1.4)	Portal vein or hepatic vein	(0-128)	1. Pringle maneuvers 2. Cirrhotic livers 3. Vessels smaller than 3 mm in diameter
Chang et al. [22]	2012	Korea	NA	Primary	1(2.6)	Portal vein	NA	Cirrhotic livers
Koda et al. [23]	2012	Japan	NA	Primary	32(0.2)	Portal vein	NA	NA
Desolneux et al. [24]	2014	France	NA	Secondary	3(2.0)	Portal vein or hepatic vein	NA	NA
Fang et al. [25]	2014	China	NA	Primary	1(0.8)	Portal vein	NA	NA
Kai et al. [26]	2015	China	NA	Primary	1(2.9)	Portal vein	NA	NA
Singh et al. [27]	2016	England	69/M	Secondary	1(NA)	Hepatic vein	30	Tumors close to the vein
Hairol et al. [28]	2017	Korea	66/M	Secondary	1(NA)	Portal vein	14	1. Cirrhotic livers 2. Hypercoagulative states
Verloh et al. [29]	2019	Germany	NA	Primary	1(0.9)	Portal vein	NA	NA
Maeda et al. [30]	2020	Japan	NA	Primary	35(0.4)	Portal vein	NA	NA

Abbreviations: PBO percutaneous balloon occlusion; NA not available; MWA microwave ablation; RFA radiofrequency ablation

Finally, the main means of preventing IVC thrombosis are as follows: (1) An increase in the frequency of MRI or CT detection is recommended within 2 months. Ah Yeong Kim et al. found that the mean time of hepatic vein thrombus formation after RFA was 37 days [21]. In our case, no hepatic vein thrombosis was found one month after MWA, but hepatic vein and IVC thrombosis were accidentally found two months after the operation (the patient’s D-dimer level was normal). (2) When a patient is diagnosed with hepatic venous thrombosis, anticoagulation therapy is recommended to prevent thrombus progression and embolus shedding. Although it was reported that the vast majority of hepatic thromboses had no clinical symptoms, a few had poor outcomes because of portal hypertension or hepatic failure [14, 15]. Therefore, if the formation of suprahepatic vena cava thrombosis is not treated in time, the patient may develop symptoms of pulmonary embolism, which can be fatal.

## Conclusion

In conclusion, thrombosis in the suprahepatic IVC is extremely rare, and accurate imaging diagnosis followed by thrombolysis and anticoagulation therapy can prevent fatal complications.

## Data Availability

Not applicable.

## Abbreviations

IVC: Inferior vena cava
PTE: Pulmonary thromboembolism
CRC: Colorectal cancer
CRLM: Colorectal liver metastases
CRM: Circumferential resection margin
MWA: Microwave ablation
RFA: Radiofrequency ablation
